# Comparative Analysis of the EF-1α Intergenic Region in *Babesia divergens* Isolates: Insights into TA Repeat Variation and Potential Regulatory Implications

**DOI:** 10.3390/ijms27052222

**Published:** 2026-02-26

**Authors:** Sezayi Ozubek, Alejandro Sanchez-Flores, Estrella Montero, Heba Alzan, Carlos E. Suarez, Ricardo Grande, Aitor Gil, Munir Aktas, Luis Miguel González

**Affiliations:** 1 Department of Parasitology, Faculty of Veterinary Medicine, University of Firat, 23119 Elazig, Türkiye; sezayi.ozubek@wsu.edu (S.O.); maktas@firat.edu.tr (M.A.); 2 Unidad Universitaria de Secuenciación Masiva y Bioinformatica, Institute of Biotechnology, Universidad Nacional Autónoma de México (UNAM), Cuernavaca CP 62210, Mexico; ricardo.grande@ibt.unam.mx; 3 Laboratorio de Referencia e Investigacion en Parasitologıa, Centro Nacional de Microbiología, ISCIII Majadahonda, 28220 Madrid, Spain; aitor.gil@externos.isciii.es (A.G.); lmgonzal@isciii.es (L.M.G.); 4 Parasitology and Animal Diseases Department, National Research Center, Dokki, Giza 12622, Egypt; heba_alzan@wsu.edu; 5 Department of Veterinary Microbiology and Pathology, College of Veterinary Medicine, Washington State University, P.O. Box 647040, Pullman, WA 99164, USA; suarez@wsu.edu

**Keywords:** *Babesia divergens*, comparative analysis, Elongation Factor 1 alpha (EF-1α), host range

## Abstract

*Babesia divergens*, a zoonotic tick-borne pathogen, causes bovine and human babesiosis in Europe. The Elongation Factor 1 alpha (EF-1α) protein is important in many cellular processes and has emerged as a possible target for subunit vaccine development against parasitic infections, and its intergenic region (IG) is an important tool for genetic manipulation of *Babesia* parasites. While the EF-1α locus of *B. divergens* has been described, structural variation between isolates was poorly defined. In order to fill this gap, we performed a comparative analysis of the EF-1α-IG in *B. divergens* human (Rouen 87 and Spanish sample) and bovine (Türkiye) host isolates. Our findings revealed both conserved and variable elements, particularly in TA nucleotide repeat numbers and IG sequence length. The Spanish isolate exhibited the highest TA repeat expansion, whereas the Rouen 87 strain had the shortest IG. Given the known role of repeat-rich promoter elements in gene regulation, these differences may influence EF-1α transcription. Additionally, these findings provide insights into the evolutionary divergence of *B. divergens* and its host adaptation mechanisms. This study establishes a foundation for future gene editing and transfection strategies, where selecting intergenic sequences with varying TA repeats could optimize transfection efficiency and explain phenotypic differences between isolates from different hosts or regions.

## 1. Introduction

Elongation Factor 1 alpha (EF-1α) is among the most abundant proteins ever reported to be expressed by eukaryotic cells and is highly conserved [1,2,3,4]. Traditionally, EF-1α is chiefly acknowledged for its contribution to the translation machinery, facilitating the binding of aminoacyl-*tRNA* to the ribosomal A site in a GTP-dependent manner [5]. Nevertheless, recent studies have revealed how it functions in various cellular processes such as microtubule severing, ubiquitin-dependent proteolysis of N-terminal blocked proteins and cytoskeletal rearrangements [3,6]. In addition, EF-1α plays a crucial role in fundamental cellular events, including programmed apoptosis, in which it is up-regulated [3,7,8]. Further studies have also highlighted the important role of EF-1α in other apicomplexan parasites, including *Cryptosporidium parvum* Tyzzer, 1912, and *Toxoplasma gondii* (Nicolle & Manceaux, 1908), for the entry process of these parasites into host cells. This suggests that EF-1α could be a promising target for vaccine development against cryptosporidiosis and toxoplasmosis [9,10]. Furthermore, results from studies on vaccination against avian coccidiosis indicate that EF-1α is a promising candidate antigen for eliciting cross-protective immunity against this disease in poultry, highlighting its potential for veterinary use [11].

*Babesia* are *T. gondii*- and *Plasmodium*-related apicomplexan tick-borne parasites responsible for acute and persistent infections in vertebrate hosts, including cattle and humans. Advancements in gene editing and transfection systems are essential for unraveling the molecular intricacies of *Babesia* parasites and enhancing vaccine development strategies [12,13]. Among the crucial components, facilitating these advancements is the EF-1α, which plays a pivotal role in various cellular processes. In both *Plasmodium* and *Babesia* parasites, the *EF-1α* gene exhibits a distinctive organization, featuring two identical head-to-head genes, each separated by intergenic regions [14]. The EF-1α intergenic region (EF-1α-IG) hosts robust promoters capable of driving the expression of both *EF-1α* genes efficiently. This unique characteristic of the EF-1α-IG also enables it to effectively promote the expression of foreign genes in transiently and stably transfected *Babesia bovis* and *Babesia bigemina* parasites [12]. The utilization of these strong promoters within the EF-1α-IG has dramatically accelerated the development of transfection and gene editing systems in both *Plasmodium* and *Babesia* parasites [12,15]. Researchers have successfully exploited these promoters to introduce foreign genes into parasite genomes, facilitating precise genetic manipulations, the development of novel vaccines, and sophisticated studies on parasite biology. This breakthrough has significantly improved gene function analysis, advanced our understanding of the molecular mechanisms underlying parasite pathogenesis and host–parasite interactions, and has become an important tool for developing novel vectored vaccines [13,16].

*Babesia divergens*, one of the three major species of *Babesia* causing babesiosis in cattle, is particularly significant as a zoonotic pathogen playing a role in human babesiosis in Europe, in addition to its impact on cattle [17,18]. Compared to *B. bovis* and *B. bigemina*, limited information is available about the infectivity and pathogenicity of *B. divergens*, leaving many questions unanswered regarding important processes. Understanding the gaps of knowledge on gene function and other mechanisms involved in the biology of *B. divergens* will likely require the identification of novel promoters that can aid in the development of gene editing and transfection systems. Thus far, only one study has drawn attention in this regard, describing a stable transfection system for *B. divergens* based on the promoter of the *elongation factor Tu GTP binding domain family protein* (*ef-tgtp*) gene [19]. Unlike other *Babesia* species found in cattle, *B. divergens* has a broader host spectrum, including humans. We hypothesize that the EF-1α locus of *B. divergens*, a parasite able to infect bovine and human hosts, exhibits structural and regulatory variations among different isolates, which may be linked to its host adaptability, evolutionary divergence, and geographic distribution. Investigating these differences will enhance our understanding of *B. divergens* biology and provide insights into potential mechanisms of gene regulation and parasite-host interactions.

Here, based on existing genome data and NGS and Sanger sequencing, we focused on conducting a comparative analysis of the EF-1α locus from *B. divergens* isolates obtained from humans and cattle in different geographical regions, using the *B. divergens* Rouen 87 (R87) genome as a reference [20]. Our approach focused on conducting a comparative analysis of the EF-1α locus, which serves as a pivotal component in transfection investigations, among *B. divergens* isolates sourced from infected bovines in Türkiye and from patients with severe babesiosis in Spain [21]. The data generated in this study imply the possible influence of evolutionary selective forces shaping key sequence and structural differences in the EF-1α locus between these two isolates.

## 2. Results

### 2.1. EF-1α Locus Organization and Synteny in Babesia divergens

**Figure 1:**
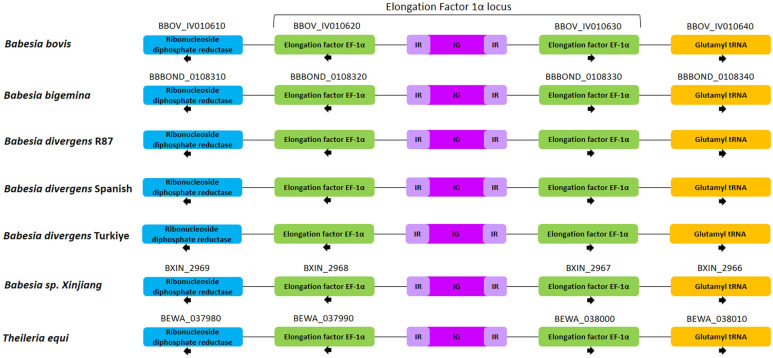
Schematic representation of gene localization and synteny maps of the *EF-1α* gene locus. IR: Inverted repeat; IG: Intergenic region.

In apicomplexan parasites, the *EF-1α* gene locus predominantly resides between the Ribonucleoside diphosphate reductase and Glutamyl tRNA genes, as demonstrated by conserved synteny between *Theileria* and *Babesia* species. Like *B. bovis*, *B. bigemina*, *Babesia* sp. Xinjiang and *Theileria equi*, the *B. divergens* R87, Spanish and Türkiye isolates also possess two identical *EF-1α* genes located within the *EF-1α* locus (Figure 1).

### 2.2. EF-1α Coding Sequence Conservation Across Babesia divergens Isolates

**Figure 2:**
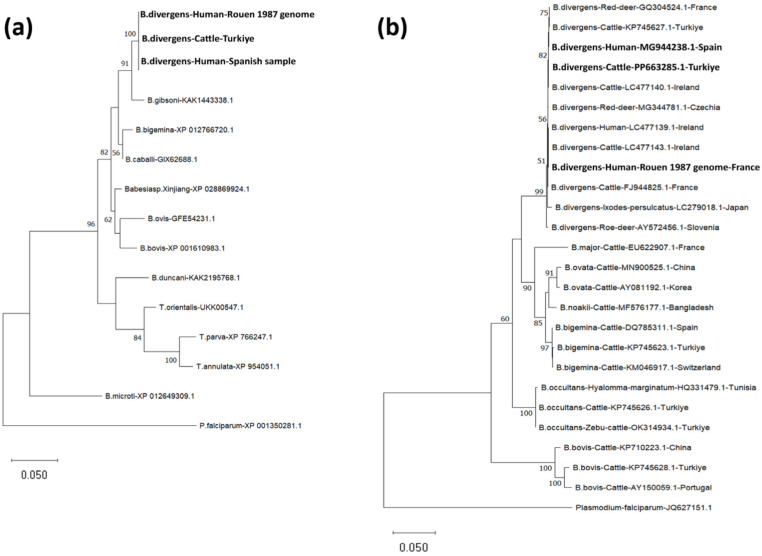
(**a**) Phylogenetic analyses of piroplasm EF-1α protein sequences by maximum likelihood method based on the Le_Gascuel_2008 model [22]. Sequences were obtained from the GenBank database. The EF-1α protein sequence of *Plasmodium falciparum* was utilized as an outgroup. (**b**) Phylogenetic analyses of piroplasm *18S rRNA* gene sequences by the maximum likelihood method based on the Tamura-Nei model [23]. The *18S rRNA* gene sequence of *P. falciparum* was utilized as an outgroup. *Babesia divergens* R87, Spanish, and Türkiye isolates of EF-1α are highlighted in bold.

Comparative analysis of the EF-1α locus in *B. divergens* isolates (R87, Spanish, and Türkiye isolates) revealed that the EF-1α gene sequences (1347 bp) were 100% identical across all isolates. Each of the two EF-1α ORFs from three isolates of *B. divergens* encodes identical proteins of 448 amino acids, with an estimated molecular mass of 49.39 kDa, which is the same size as those encoded by *B. bovis* (XP_001610983.1), *B. bigemina* (XP_012766720.1), *Babesia caballi* (GIX62688.1), *Babesia gibsoni* (KAK1443338.1), *Babesia duncani* (KAK2195768.1), *Babesia ovis* (GFE54231.1), *Babesia* sp. Xinjiang (XP_028869924.1), *Theileria parva* (XP_766247.1), *Theileria annulata* (XP_954051.1), and *Theileria orientalis* (UKK00547.1). The EF-1α *B. divergens* R87, Spanish, and Turkiye ORFs show identical sequences (100% identity) and share significant similarity with homologous sequences found in *B. gibsoni* (97.99% identity), *B. caballi* (97.54% identity), *B. bigemina* (96.65% identity), *Babesia* sp. Xinjiang (96.43% identity), *B. bovis* (94.87% identity), *B. ovis* (94.20% identity), *B. duncani* (93.97% identity), *T. orientalis* (92.41% identity), *T. parva* (89.51% identity), *T. annulata* (89.06% identity), and *B. microti* (86.58% identity) (Figure S1). Pairwise amino acid comparisons with mammalian homologs showed ~73.38% identity to human EF-1α (*Homo sapiens*, NP_001393.1) and bovine EF-1α (*Bos taurus*, NP_776960.1) (Figure S1). The human and bovine EF-1α sequences were identical in this analysis (100% identity). Notably, key regions involved in guanosine 5′-triphosphate (GTP) binding are conserved across these organisms, as depicted in Figure S2. A phylogenetic analysis using the EF-1α protein sequence of R87, Spanish, and Türkiye isolates, along with other related piroplasma species, showed that both isolates clustered within the same clade as *B. gibsoni* (Figure 2a). Similarly, in the phylogenetic analysis of *B. divergens* R87, Spanish (MG944238.1), and Türkiye (PP663285.1) *18S rRNA* gene sequences, it was observed that they were in the same clade with *B. divergens* isolates obtained from different hosts from GenBank (Figure 2b).

### 2.3. Features, Sequence Alignment and Nucleotide Variability in IG

**Figure 3:**
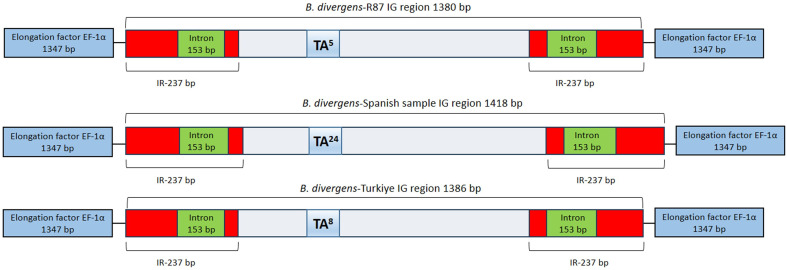
Schematic representation of the EF-1α-IG between *B. divergens* isolates. The terminal inverted repeat (IR) regions (red), intron (green, 153 bp), and TA repeats (blue) are highlighted. The IG length varies due to differences in TA repeat numbers, with five TA repeats in R87 (1380 bp), 24 in the Spanish isolate (1418 bp), and eight in the Türkiye isolate (1386 bp).

The EF-1α-IGs of the three distinct *B. divergens* isolates analyzed here are not related in sequence to the IGs of other related *Babesia* parasites. However, the *B. divergens* EF-1α-IGs do have structural similarities with other *Babesia* parasites, including the presence of terminal inverted repeat (IR) regions of 237 bp, which were conserved among all isolates. In addition, as found in *B. bovis* and *B. bigemina* [12], the IR regions harbor a 153 bp intron, maintaining sequence integrity across isolates (Figure 3).

However, the comparative analysis of the EF-1α locus sequences in the three *B. divergens* isolates (R87, Spanish, and Türkiye isolates) revealed sequence differences in the IG. While the EF-1α gene sequences were found to be 100% identical among all isolates, the IG length varied across the isolates, with the R87 strain containing five TA repeats and measuring 1380 bp, the Spanish isolate harboring 24 TA repeats with an IG of 1418 bp, and the Türkiye isolate exhibiting eight TA repeats with an IG of 1386 bp (Figure 3). Overall, these findings indicate notable differences in the IG between *B. divergens* isolates originating from different geographical regions and host sources.

**Figure 4:**
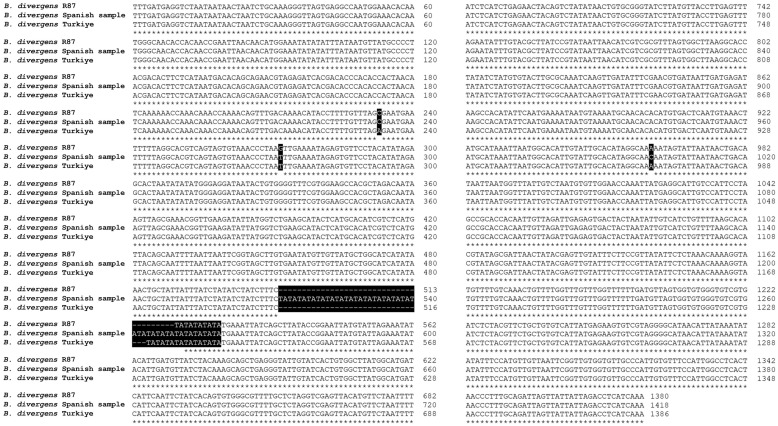
Multiple sequence alignment of the IG within the EF-1α locus between *B. divergens* isolates. Nucleotide variations are highlighted with black boxes, while differences in TA repeat numbers are also marked. “*”= identical nucleotides.

Multiple sequence alignment of the IG across the three *B. divergens* isolates further confirmed variations in TA repeat numbers and additional nucleotide substitutions. The TA repeat sequences were located around position ~513–516 bp across all isolates but varied in number. Besides TA repeat variations, additional sequence polymorphisms were identified across the IG (Figure 4). The results were consistent and validated using Nanopore and Sanger sequencing technologies.

In addition to *B. divergens*, the intergenic region was examined in other zoonotic *Babesia* species, including *Babesia* MO1 and *B. duncani*. While the EF-1α locus in *B. duncani* lacked TA repeats, the *Babesia* MO1 genome contained a short stretch of three TA repeats. These findings indicate that the presence and expansion of TA repeats may be specific to *B. divergens* (Figure S3).

### 2.4. Validation of TA Repeat Variations in the IG

**Figure 5:**
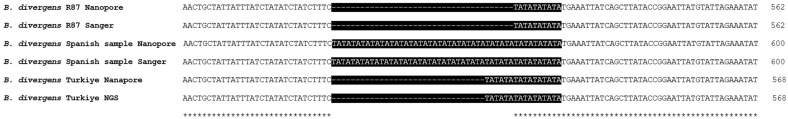
Multiple sequence alignment of the TA repeat region across *B. divergens* isolates using Nanopore, Sanger, and NGS sequencing methods. The black-highlighted regions indicate TA repeats. “*”= identical nucleotides.

To confirm the sequence differences in the TA repeat region among *B. divergens* isolates, a combination of PCR amplification, Sanger sequencing, and NGS approaches were employed. For the R87 isolate, the IG was amplified using the Bdiv-Ef-For1/IGRev1 primer pair, yielding an amplicon of approximately 2.8 kb, which was subsequently validated by Sanger sequencing. The repeat structure identified in the sequencing data confirmed the presence of five TA repeats, consistent with initial findings. For the Türkiye isolate, the IG was amplified in two overlapping fragments using the Bdiv-Ef-For1/Sez-For1 and Sez-For1-Rev/GlutamylR1 primer sets. These amplicons were subjected to NGS analysis, which verified the eight TA repeats and confirmed the overall sequence integrity of the IG. For the Spanish isolate, initial attempts to amplify the full IG using Bdiv-Ef-For1/IGRev1 primers were unsuccessful, likely due to sequence divergence at the primer binding sites. An alternative primer pair, IGLMFR1/IGRev1, was used to amplify a ~1 kb fragment, which was validated through Sanger sequencing. The results confirmed the presence of 24 TA repeats, aligning with the Nanopore sequencing data (Figure 5).

## 3. Discussion

The EF-1α-IG contains bi-directional promoters capable of efficiently driving expression of *EF-1α* genes. Their utilization has significantly advanced transfection systems in *Babesia* [12]. In this study, we explored the structural and sequence variability of the EF-1α-IGs across three *B. divergens* isolates from humans and cattle to better understand their potential role in gene regulation and parasite adaptation. The findings indicated that while the EF-1α coding sequences remain highly conserved, there is variability in the IG, specifically in TA repeat numbers, suggesting that these elements could contribute to transcriptional regulation and genomic stability. According to the model proposed by Uhlemann et al. (2004) [24], the expression of NADPH oxidase is influenced by a highly polymorphic TA repeat region located ~550 bp upstream of the gp91phox promoter. Variation in the number of TA repeats has been associated with reduced promoter activity and enzyme levels, as well as protection against severe malaria. This demonstrates that these repeats can generate functionally relevant differences in gene expression and disease outcome. Therefore, TA repeat polymorphisms should be considered functional regulatory elements that can modulate transcriptional efficiency and potentially influence host–parasite adaptations and interactions. As these repeats are situated in promoter-proximal regulatory regions upstream of strong promoters, such as EF-1α, their presence may impact transcriptional efficiency by altering promoter architecture.

The high level of conservation of the *EF-1α* gene observed in different strains of *B. divergens* and its similarity to the EF-1α protein of the two other pathogenic bovine babesiosis species, *B. bovis* and *B. bigemina*, highlights the potential use of EF-1α in subunit vaccines [12]. These findings revealed that the EF-1α protein has the potential to be a suitable antigen for vaccine development, as it was previously suggested for other important protozoa such as *T. gondii* [10] and *C. parvum* [9], which are also members of the apicomplexan phylum. Additionally, recombinant EF-1α has been reported to elicit cross-protective immunity against *Eimeria tenella* and *Eimeria maxima* in broiler chickens [11], highlighting its immunogenic potential in apicomplexan parasites. While this finding does not demonstrate cross-immunity among *Babesia* species, it supports the idea that recombinant EF-1α may be a broadly immunogenic antigen. However, our sequence comparisons highlight that EF-1α is highly conserved between piroplasmids and mammalian hosts, so it should be considered cautiously as a vaccine antigen. Any vaccine-focused development will require experimental work to identify parasite-specific epitopes and to evaluate potential host cross-reactivity.

Comparisons of the IGs among the three *B. divergens* isolates (R87, Spanish, and Türkiye isolates) provided novel insights into the structural organization of this region. This study revises the common expectation, largely based on observations from *B. bovis* and *B. bigemina*, that EF-1α intergenic/promoter regions are highly conserved across *Babesia* spp. and among isolates. Importantly, this conservation had not been systematically examined in *B. divergens*. Despite having identical *EF-1α* gene ORFs organized in a typical head-to-head fashion, *B. divergens* isolates exhibited striking differences in their IGs, despite sharing identical *18S rRNA* sequences. One of the most unexpected findings of this study was the variability in TA repeat numbers within the IG. The Spanish isolate exhibited the highest number of TA repeats (*n* = 24), while the Türkiye isolate contained eight, and the R87 strain had only five. Given that repeat sequences in intergenic regions can affect promoter activity, transcription factor binding, and chromatin accessibility [25], these differences may influence *EF-1α* gene expression. TA-rich repeats may reshape local DNA structure, which could influence promoter function. Because TA-repeat length differs between isolates, the regulatory context may vary, potentially leading to differences in EF-1α expression levels. The conserved 237 bp terminal IR region and the 153 bp intron within the untranslated region suggest that the IG may have multiple regulatory roles. Inverted repeats (IRs) are often associated with promoter function and transcriptional regulation, while intronic sequences within untranslated regions can influence alternative splicing, mRNA stability, or post-transcriptional control. The conservation of this structure across isolates suggests its functional importance in *EF-1α* gene regulation.

As mentioned, the differences within the IGs among *B. divergens* isolates analyzed hereby predominantly occur in the initial segment of the putative promoter regions. Although investigations into EF-1α-IG variations across different strains remain limited, examination of these regions from *B. bovis* strains in Argentina, Mexico, and Australia suggests a notable degree of conservation among them. The distinctions between *B. bovis* and *B. divergens*, despite both being etiological agents of babesiosis in cattle, are evident in their geographical distribution, different tick vector preferences, and the clinical manifestations of babesiosis [18]. Moreover, the broader host spectrum of *B. divergens*, including its zoonotic potential, highlights its adaptability and significance beyond the confines of bovine hosts. It is thus reasonable to speculate that the discrepancies observed in the EF-1α-IG may be influenced by host-associated factors, although this remains a hypothesis and has not been directly tested in the present study. This is particularly noteworthy in our study, where R87, Spanish, and Türkiye isolates, whose elongation loci we compared, were obtained from humans (R87 and Spanish isolates) and cattle (Türkiye), respectively. Surprisingly, the EF-1α-IG from parasites that infect humans presented differences that could also be related to virulence or adaptation to laboratory conditions. Such findings raise intriguing questions regarding factors involved in parasite evolution and the potential influence of host-specific factors on the genetic makeup and regulatory mechanisms of *Babesia* species, warranting further investigation into the intricate interplay between pathogen genetics and host environments. As these findings revise prior expectations, the substantial differences found in the IGs of the *EF-1α* genes between three different strains of *B. divergens*, as confirmed by *18S rRNA* gene analysis, are reported for the first time among piroplasmid parasites. Moreover, as previously noted, these differences may reflect important adaptive events affecting the development of the parasites among distinct invertebrate and/or vertebrate hosts.

Surprisingly, upon expanding our comparative analysis of the EF-1α-IGs found in other zoonotic *Babesia* species, *B. duncani* and *Babesia* MO1, striking differences were also noted in their EF-1α-IGs. *Babesia duncani*, on the other hand, contained no TA repeats, while only three TA motifs were present in the MO1 isolate. This suggests that TA repeat expansion within the EF-1α-IG may represent a species-specific regulatory feature rather than a common trait of all zoonotic *Babesia*. Further comparative studies across more isolates will be essential to determine whether these differences contribute to host tropism, gene expression variability, or virulence mechanisms.

## 4. Materials and Methods

### 4.1. Babesia divergens—Rouen 87 Strain (R87)

*Babesia divergens* R87 is a reference strain originally isolated from a human case of babesiosis in France [26]. This strain has been widely used in molecular and genomic studies due to its well-characterized genome sequence [20]. The EF-1α locus data from this strain were utilized in this study and were obtained from the *B. divergens* genome assembly described by [20].

### 4.2. Babesia divergens Spanish Sample

*The Babesia divergens* Spanish strain was isolated from a blood sample of an elderly patient from Asturias, Spain, who suffered a fulminant fatal babesiosis [21]. The blood sample was cultured in vitro in human A+ RBCs in RPMI 1640 (Gibco, Grand Island, NY, USA) supplemented with 10% human serum (The Interstate Companies, Memphis, TN, USA), 7.5% (*w*/*v*) sodium bicarbonate solution (Lonza Group Ltd., Basel, Switzerland, 144-55-8), and 100 μmol/L hypoxanthine (Sigma-Aldrich Corporation, St. Louis, MO, USA, H9377) at a pH of 7.3. Cells were cultured at 37 °C in a humidified atmosphere of 5% CO_2_. The culture medium was replaced every 24 h, and parasitemia was monitored by examining Giemsa-stained blood smears under a light microscope.

### 4.3. Babesia divergens Türkiye Sample

*The Babesia divergens*-Türkiye isolate was obtained from a 3-year-old cow exhibiting severe babesiosis symptoms, including high fever, anemia, and hemoglobinuria, in Bartin Province of Türkiye in 2023. Microscopic examination revealed a parasitemia level of 1.5%. Following the collection of blood stabilate from the animal, treatment was administered using imidocarb dipropionate [18]. To determine the species of *Babesia*, Reverse Line Blot (RLB) analysis was conducted. Total DNA extraction from blood samples was performed using the PureLink™ Genomic DNA Mini Kit (Invitrogen Corporation, Carlsbad, CA, USA) according to the manufacturer’s instructions. The hypervariable V4 region of the piroplasm *18S rRNA* gene was then amplified using primers RLB-F2 and RLB-R2-biotin [27], specifically for the RLB assay. RLB was carried out on the PCR product following the previously described method [28]. After amplification, 20 µL of PCR products obtained from each DNA sample were diluted to a final volume of 150 µL with 2X SSPE/0.1% SDS buffer. For RLB hybridization, the samples were heated at 95–100 °C for 10 min in a Thermal Cycler and rapidly cooled on ice. Subsequently, the PCR products were hybridized with probes specific to the genera and species of *Babesia* and *Theileria*, which were linked to an RLB membrane [28] (Table S2).

### 4.4. Comparative Analysis of EF-1α in Babesia MO1 and Babesia duncani

In this study, we used the EF-1α locus data of these species obtained from the *Babesia* MO1 [29] and *B. duncani* [30,31] assemblies. Specifically, Bioproject PRJNA1032622 for *Babesia MO1* and Bioproject PRJNA821606 and locus OL804102 for *B. duncani* were used. To assess the presence of TA repeats in *Babesia* MO1 and *B. duncani*, we retrieved the EF-1α locus from *Babesia* MO1 and *B. duncani* genome assemblies (PRJNA1032622 and PRJNA821606, respectively). The intergenic regions were identified by locating the flanking genes (*ribonucleoside diphosphate reductase* and *glutamyl tRNA synthetase*) and were subsequently aligned to detect potential tandem repeat regions. The presence or absence of TA repeats was manually inspected using multiple sequence alignment.

### 4.5. Designing Primers for the EF-1α Locus and PCR Amplification

Primers were designed utilizing sequences from *Ribonucleoside diphosphate reductase* (Bdiv_023390c) and *Glutamyl tRNA* (Bdiv_023400) genes obtained from the Piroplasma DB (https://piroplasmadb.org) and nanopore sequencing data to amplify the EF-1α locus. All primers were designed using the Primer Quest™ Tool (https://www.idtdna.com/pages/tools/primerquest, accessed on 9 May 2023) (Table S1 and Figure S4). PCR amplification was carried out using Phusion^®^ High-Fidelity PCR Master Mix with GC Buffer (M0532S; New England Biolabs, Ipswich, MA, USA). The PCR was performed in a total reaction volume of 20 μL containing 10 μL of 2X Phusion Master Mix, 1 μL of each forward and reverse primer, 1 μL of template DNA, and 7 μL of nuclease-free water. A no-template control (NTC), in which nuclease-free water was used instead of template DNA, was included in each PCR run to monitor for contamination.

### 4.6. Nanopore Sequencing of Babesia Genomic DNA

The total DNA from *Babesia* samples was used to prepare Oxford Nanopore libraries with the SQK-LSK114 Ligation Sequencing Kit for 24 h in the MinION Platform with an R10.4 flow cell, following the manufacturer’s protocol (Oxford Nanopore Technologies, Oxford, UK). The base calling was performed using the software Dorado v0.8.3 with the duplex option. The sequencing and basecalling were carried out at the Unidad Universitaria de Secuenciación Masiva y Bioinformática, Instituto de Biotecnología, Universidad Nacional Autónoma de México (Cuernavaca, Mexico).

### 4.7. Illumina Sequencing and De Novo Assembly

A sequencing library was prepared using the Nextera XT DNA Library Preparation Kit (Illumina, Inc., San Diego, CA, USA), and sequencing was performed using the Illumina Miseq platform (Illumina, Inc., San Diego, CA, USA) as paired-end (PE) 2 × 150 base reads. Raw NGS reads (FASTQ) were quality checked using FASTQC [32] and trimmed by Trimmomatic v0.32 [33]. Demultiplexing and low-quality read filtering were performed via CLC Genomics Workbench (Qiagen, Hilden, Germany). The de novo assembly constructed by the CLC Genomic’s de novo assembly module with the parameters of minimum contig size of 200 bp, a mismatch cost of 2, an insert cost of 3, a deletion cost of 3, a length fraction of 0.5, a similarity fraction of 0.8, and paired read input with a minimum distance of 180.

### 4.8. Sanger Sequencing

To validate the assembled EF-1α-IG, Sanger sequencing was performed on PCR-amplified IGs from the *B. divergens* isolates. PCR products were purified using the mi-Gel Extraction Kit (Metabion international AG, Steinkirchen, Germany) and sequenced using an ABI PRISM 3730XL DNA Analyzer (Applied Biosystems, San Francisco, CA, USA). The resulting chromatograms were manually inspected, corrected, and edited using the Chromas Pro program v2.6.6 (Technelysium Pty Ltd., Brisbane, Australia) and the Laser Gene 12.1 program (DNAStar, Madison, WI, USA), and the sequences were aligned against the de novo assembled IG to confirm accuracy and resolve potential misassembles.

### 4.9. Phylogenetic Analysis

Phylogenetic relationships were inferred using Molecular Evolutionary Genetics Analysis (MEGA 11) [34]. The maximum likelihood (ML) method was employed to construct phylogenetic trees based on EF-1α protein sequences and *18S rRNA* gene sequences from various piroplasm species. Bootstrap support values were calculated with 1000 replicates to assess tree robustness.

### 4.10. Ethical Statement

*B. divergens* DNA samples from cattle were obtained as part of routine veterinary diagnostic procedures from a clinically infected animal. No experimental infection or additional intervention was performed for research purposes. Therefore, Institutional Animal Care and Use Committee (IACUC) or Ethics Committee approval was not required. The *B. divergens* Spanish strain was obtained as part of routine diagnostic procedures; no additional ethics approval or informed consent from hospitals or patients was required, as our study does not involve human subjects, identifiable human tissue, or personal data, in accordance with the Declaration of Helsinki (Research Ethics Committee, Instituto de Salud Carlos III, Spain, CEI PI 59_2022-v4). Human A+ blood from healthy donors was used to maintain cultures of *B. divergens* (Rouen 87). The blood and its protocol were approved for use by the Blood Transfusion Center, Madrid, Spain.

## 5. Conclusions

This study provides new insights into the structural variability of the EF-1α-IG in *B. divergens* isolates from different hosts, revealing differences in TA repeat numbers that may contribute to gene regulation and parasite adaptation. While our findings highlight the potential regulatory role of this region, functional validation through promoter activity assays, gene expression analysis, and transfection studies is still needed to determine its precise impact on EF-1α expression. Given the zoonotic nature of *B. divergens*, future research should explore whether similar variations in the IG exist in *B. divergens* isolates from cattle and humans. Additionally, the gerbil model could be used to investigate potential intergenic region variations across different vertebrate hosts to better understand the role of this region in host adaptation. Further investigation of the functional implication of these variations may provide valuable insights into transcriptional control across diverse parasite strains, provide valuable information on the mechanisms driving *B. divergens* evolution and improve transfection strategies for functional genomic studies. Overall, this study lays the groundwork for future research into gene regulation and host–pathogen interactions in zoonotic *Babesia* species.

## Data Availability

All data are provided within the manuscript or Supplementary Information Files.

## Supplementary Materials

The following supporting information can be downloaded at: https://www.mdpi.com/article/10.3390/ijms27052222/s1.

## Abbreviations

The following abbreviations are used in this manuscript:

EF-1α	Elongation Factor 1 alpha
IG	Intergenic region
EF-1α-IG	Elongation Factor 1 alpha intergenic region
*B. divergens*	*Babesia divergens*
*B. bovis*	*Babesia bovis*
*B. bigemina*	*Babesia bigemina*
*B. duncani*	*Babesia duncani*
*Babesia MO1*	*Babesia MO1*
*T. gondii*	*Toxoplasma gondii*
*C. parvum*	*Cryptosporidium parvum*
*P. falciparum*	*Plasmodium falciparum*
R87	Rouen 87 strain
TA	Thymine–Adenine (TA) nucleotide repeat(s)
GTP	Guanosine 5′-triphosphate
tRNA	Transfer RNA
ORF	Open reading frame
bp	Base pairs
kDa	Kilodalton
18S rRNA	18S ribosomal RNA
IR	Inverted repeat(s)
NGS	Next-generation sequencing
PCR	Polymerase chain reaction
kb	Kilobase(s)
RBCs	Red blood cells
RPMI	Roswell Park Memorial Institute (RPMI 1640 medium)
CO_2_	Carbon dioxide
RLB	Reverse Line Blot
DNA	Deoxyribonucleic acid
SSPE	Saline-sodium phosphate-EDTA buffer
SDS	Sodium dodecyl sulfate
μL	Microliter(s)
μmol/L	Micromole(s) per liter
IDT	Integrated DNA Technologies
NEB	New England Biolabs
PE	Paired-end
FASTQ	FASTQ sequencing file format
CLC	CLC Genomics Workbench (Qiagen software)
ABI	Applied Biosystems (ABI PRISM 3730XL)
MEGA	Molecular Evolutionary Genetics Analysis
ML	Maximum likelihood
IACUC	Institutional Animal Care and Use Committee
