# Supplementary material for: Comparative Analysis of the EF-1α Intergenic Region in Babesia divergens Isolates: Insights into TA Repeat Variation and Potential Regulatory Implications
Source: Int J Mol Sci. 2026 Feb 26;27(5):2222. doi: 10.3390/ijms27052222 (PMC12984197; doi:10.3390/ijms27052222)
Supplement: Supplementary file 1 [file ijms-27-02222-s001.zip › Supplementary Table S1.pdf]

Supplementary Table S1. Detailed information on the primers used in PCR assays.

Primer	Sequence (5'-3')
**Bdiv-Ef-For1**	TTCCCAGTCCTTCATATC
**Sez-For1**	CCGTTTCGCTAACTTATT
**Sez-For1-Rev**	AATAAGTTAGCGAAACGG
**IGFor1**	ATCTGAGAACTACAGTCTAT
**IGRev1**	TCTCATCAATTATCACGTTC
**IGLMFR1**	CCACTGTCGACGTGGCCGATAACG
**GlutamylR1**	GTGATCCACTGACTTCT
