# Supplementary material for: Comparative Analysis of the EF-1α Intergenic Region in Babesia divergens Isolates: Insights into TA Repeat Variation and Potential Regulatory Implications
Source: Int J Mol Sci. 2026 Feb 26;27(5):2222. doi: 10.3390/ijms27052222 (PMC12984197; doi:10.3390/ijms27052222)
Supplement: Supplementary file 1 [file ijms-27-02222-s001.zip › Supplementary Table S2.pdf]

Supplementary Table S2. Species-specific oligonucleotide probes used in the Reverse Line Blot (RLB) Assay.

Oligonucleotide probe	Sequence (5’-3’)
Theileria/*Babesia* genus-specific	AmMC6-TAATGGTTAATAGGA(A/G)C(A/G)GTTG
Babesia genus-specific	AmMC6-CCT(G/T)GGTAATGGTTAATAGGAA
Babesia bovis	AmMC6-CAGGTTTCGCCTGTATAATTGAG
Babesia bigemina	AmMC6-CGTTTTTTCCC TTTGTTGG
Babesia divergens	AmMC6-GTTAATATTGACTAATGTCGAG
Babesia major	AmMC6-TCCGACTTTGGTTGGTGT
Babesia occultans	AmMC6-CTCTTTTGGCCCATCTCGTCTCG
Theileria genus-specific	AmMC6-GTTGAATTTCTGCT(A/G)CAT(C/T)GC
Theileria annulata	AmMC6-CCTCTGGGGTCTGTGCA
Theileria buffeli/*orientalis*	AmMC6-GGCTTATTTCGG(A/T)TTGATTTT

AmMC6, C6 amino linker linked to the 5’
