# Supplementary figures and images for: Comparative Analysis of the EF-1α Intergenic Region in Babesia divergens Isolates: Insights into TA Repeat Variation and Potential Regulatory Implications
Source: Int J Mol Sci. 2026 Feb 26;27(5):2222. doi: 10.3390/ijms27052222 (PMC12984197; doi:10.3390/ijms27052222)

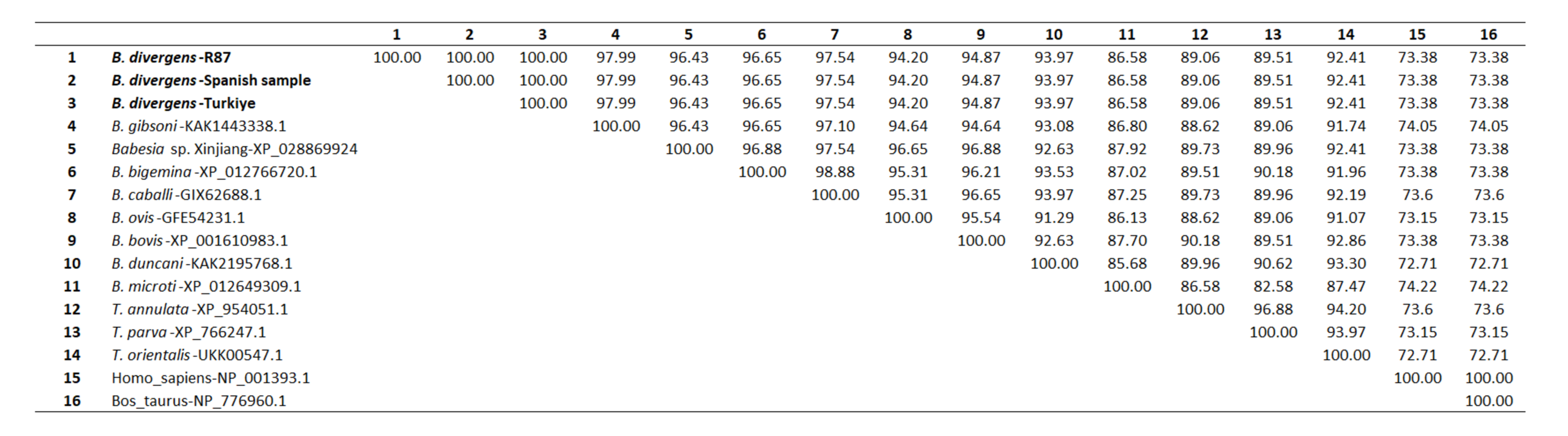

Supplement: Supplementary file 1 [file ijms-27-02222-s001.zip › Supplementary Figure S1 updated.jpg]

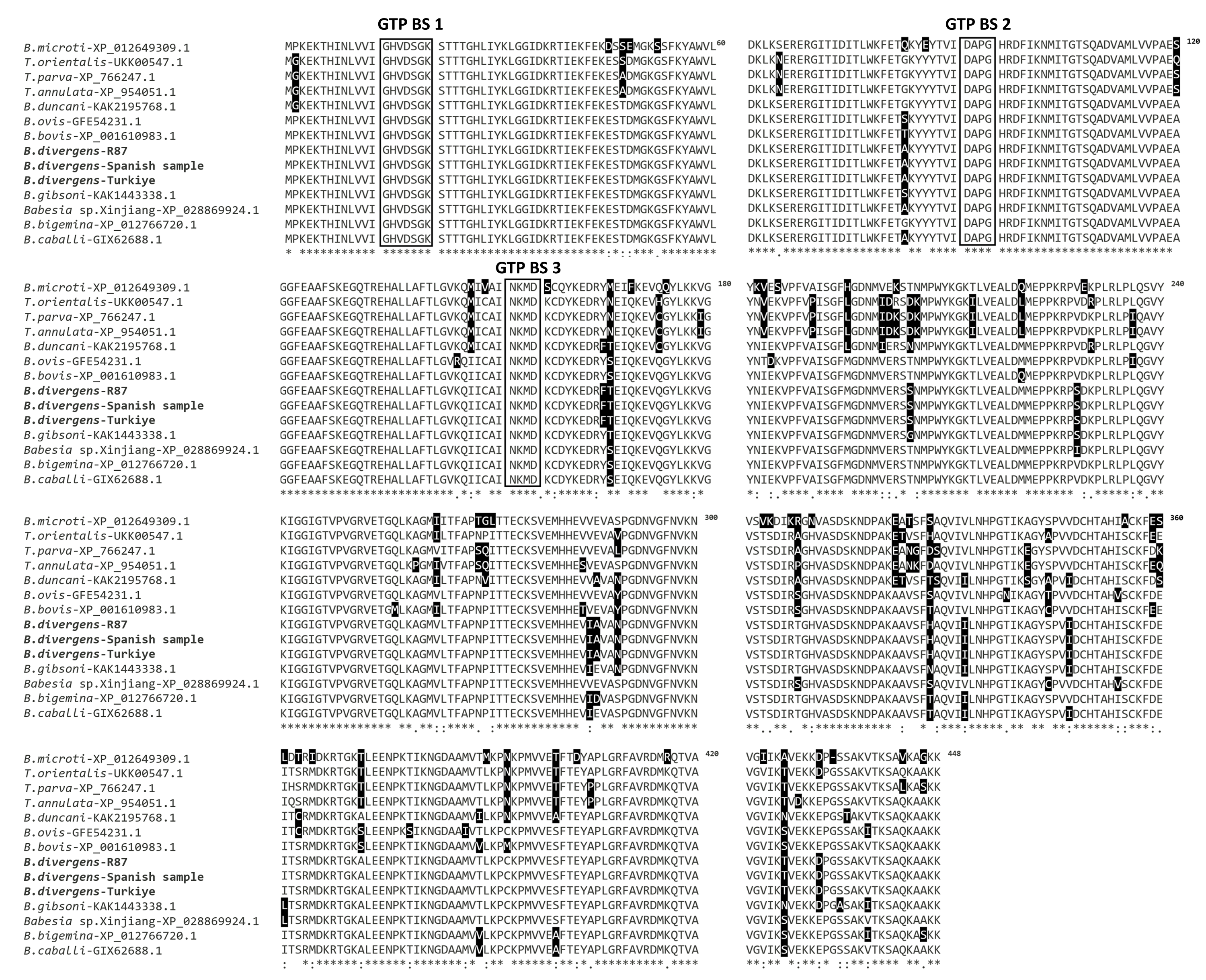

Supplement: Supplementary file 1 [file ijms-27-02222-s001.zip › Supplementary Figure S2.tif]

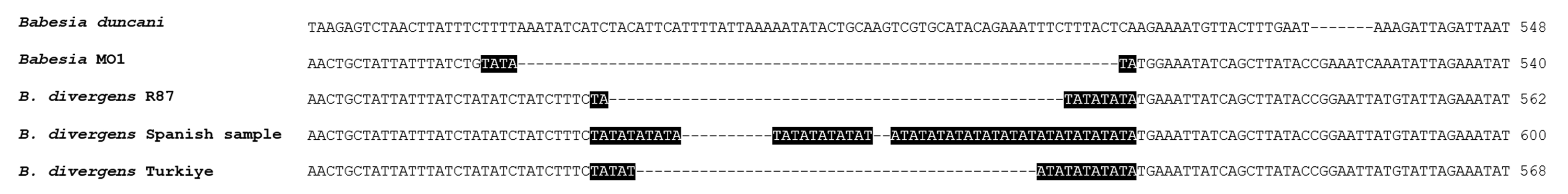

Supplement: Supplementary file 1 [file ijms-27-02222-s001.zip › Supplementary Figure S3.tif]

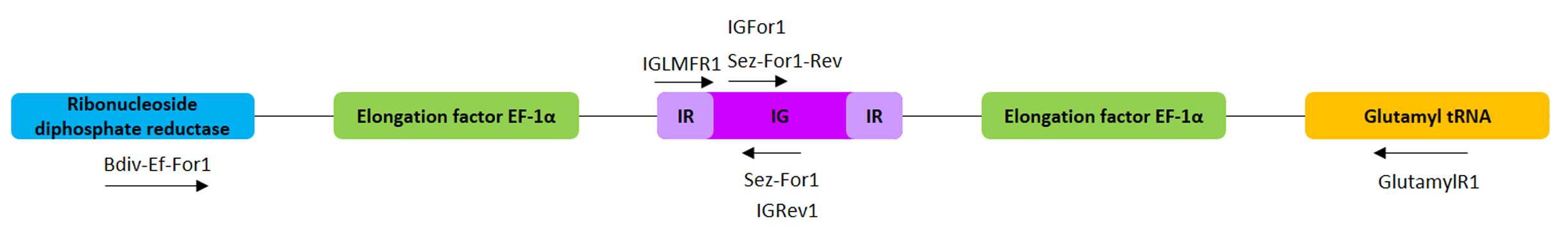

Supplement: Supplementary file 1 [file ijms-27-02222-s001.zip › Supplementary Figure S4.tif]
